# P-2098. Psychosocial Determinants and Quality of Life Among People Living with HIV in a Community Clinic Setting

**DOI:** 10.1093/ofid/ofaf695.2262

**Published:** 2026-01-11

**Authors:** Taniela M Bes, Andres E Franceschi Coll, Maysa Vilbert, Pedro A Macedo de Freitas, Sokratis Zisis, Felipe Barbosa, Thomas Treadwell

**Affiliations:** MetroWest Medical Center, Framighan, MA; Brigham and Women's Hospital, Boston, Massachusetts; MetroWest Medical Center, Framighan, MA; MetroWest Medical Center, Framighan, MA; MetroWest Medical Center, Framighan, MA; MetroWest Medical Center, Framighan, MA; MetroWest Medical Center, Framighan, MA

## Abstract

**Background:**

Despite major advances in antiretroviral therapy (ART), HIV continues to pose substantial psychosocial challenges. People living with HIV (PLWH) often experience ongoing stigma, which contributes to elevated rates of depression, social isolation, and loneliness. These factors can negatively affect treatment adherence, engagement in care, and overall health outcomes. These psychosocial burdens are not evenly distributed and disproportionately impact certain subgroups such as younger adults, migrants, and individuals with mental health diagnoses.

**Table 1:**
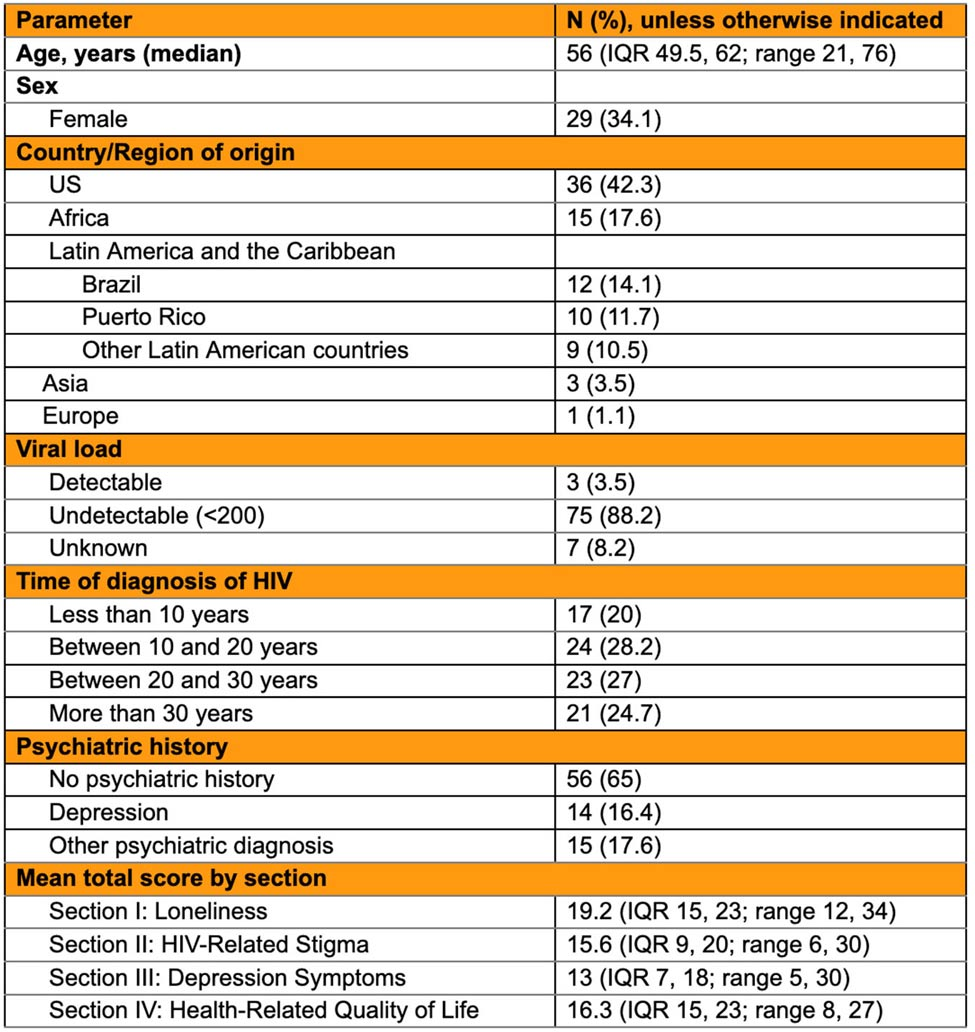
Demographics and Baseline Characteristics of the patients.

**Graphic 1:**
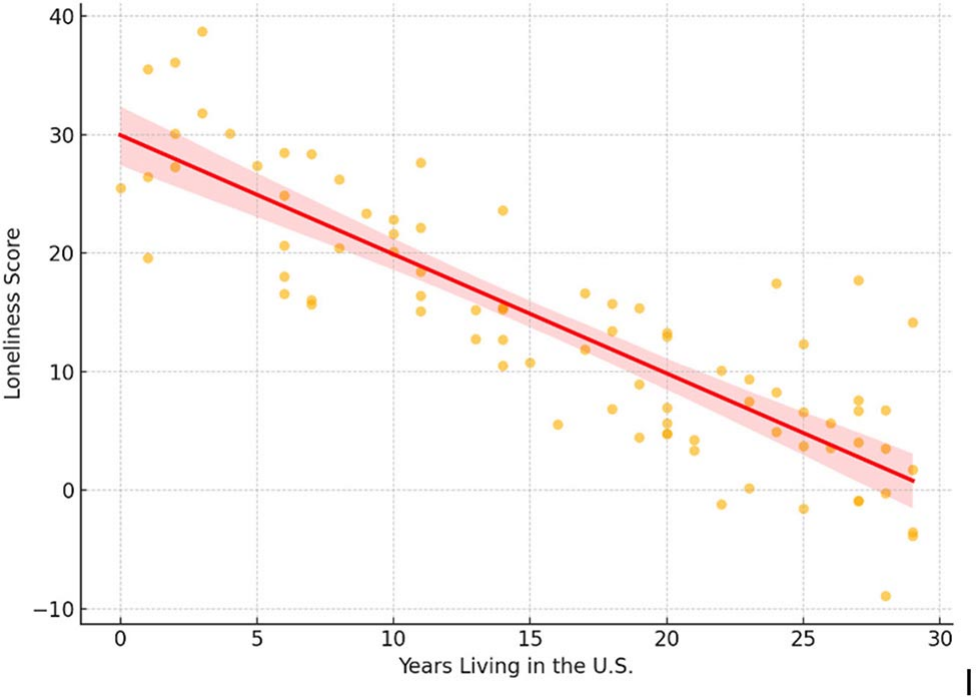
Scatter plot with regression line illustrating the relationship between years living in the U.S. and loneliness scores. A modest negative association was observed, with loneliness scores decreasing as years in the U.S. increased (Spearman’s ρ ≈ –0.25, p ≈ 0.03).

**Methods:**

This cross-sectional study, conducted at a community-based hospital in MA from November 2024 to April 2025, examined the relationships between loneliness, depression, HIV-related stigma, and demographic or clinical factors among adults diagnosed with HIV. A total of 85 participants aged 18 and older were enrolled, and 83 completed structured questionnaires assessing loneliness (via an adapted UCLA Loneliness Scale), stigma, depressive symptoms, and quality of life. Clinical data, including HIV viral load, were abstracted from medical records.

**Graphic 2:**
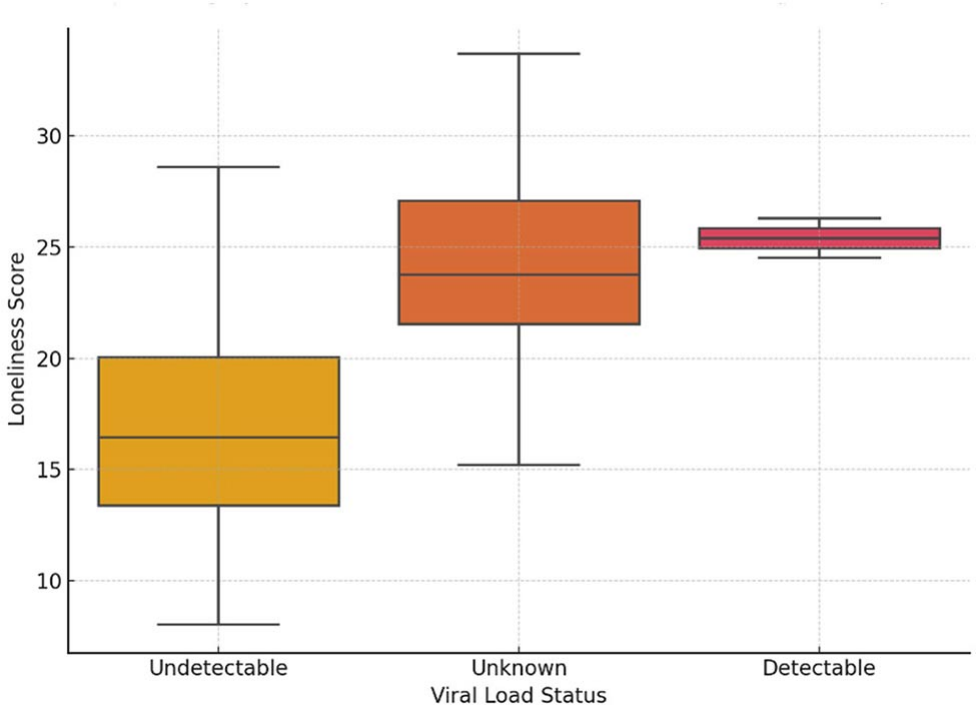
Boxplot displaying loneliness scores by HIV viral load status. Participants with unknown viral load exhibited the highest median loneliness scores. Those with undetectable viral load reported slightly lower loneliness than those with detectable levels (p = 0.040).

**Results:**

The sample was demographically diverse, including participants from the U.S., Africa, Brazil, and Puerto Rico, with a mean age of 55.5 years. Loneliness was inversely associated with age (β = –0.17, p = 0.022) and length of U.S. residence (ρ ≈ –0.25, p ≈ 0.03), suggesting younger individuals and recent migrants were more socially isolated. Participants with an unknown viral load, a potential indicator of disengagement from care, had significantly higher loneliness scores (p = 0.040). For these individuals, the viral load was unknown because they had only recently been diagnosed with HIV. Additionally, psychiatric diagnoses were strongly associated with greater depressive symptoms (β = +5.25, p = 0.011), regardless of other demographic or clinical variables.

**Conclusion:**

These findings underscore the need for integrated, community-based interventions that address loneliness and mental health in HIV care, particularly among vulnerable populations such as recent immigrants, younger adults, and those with incomplete engagement in treatment.

**Disclosures:**

All Authors: No reported disclosures

